# Feasibility of automated planning for whole‐brain radiation therapy using deep learning

**DOI:** 10.1002/acm2.13130

**Published:** 2020-12-19

**Authors:** Jesang Yu, Youngmoon Goh, Kye Jin Song, Jungwon Kwak, Byungchul Cho, Su San Kim, Sang‐wook Lee, Eun Kyung Choi

**Affiliations:** ^1^ Department of Radiation Oncology Asan Medical Center Seoul Republic of Korea

**Keywords:** whole‐brain radiation therapy, deep learning, automatic planning

## Abstract

**Purpose:**

The purpose of this study was to develop automated planning for whole‐brain radiation therapy (WBRT) using a U‐net‐based deep‐learning model for predicting the multileaf collimator (MLC) shape bypassing the contouring processes.

**Methods:**

A dataset of 55 cases, including 40 training sets, five validation sets, and 10 test sets, was used to predict the static MLC shape. The digitally reconstructed radiograph (DRR) reconstructed from planning CT images as an input layer and the MLC shape as an output layer are connected one‐to‐one via the U‐net modeling. The Dice similarity coefficient (DSC) was used as the loss function in the training and ninefold cross‐validation. Dose‐volume‐histogram (DVH) curves were constructed for assessing the automatic MLC shaping performance. Deep‐learning (DL) and manually optimized (MO) approaches were compared based on the DVH curves and dose distributions.

**Results:**

The ninefold cross‐validation ensemble test results were consistent with DSC values of 94.6 ± 0.4 and 94.7 ± 0.9 in training and validation learnings, respectively. The dose coverages of 95% target volume were (98.0 ± 0.7)% and (98.3 ± 0.8)%, and the maximum doses for the lens as critical organ‐at‐risk were 2.9 Gy and 3.9 Gy for DL and MO, respectively. The DL technique shows the consistent results in terms of the DVH parameter except for MLC shaping prediction for dose saving of small organs such as lens.

**Conclusions:**

Comparable with the MO plan result, the WBRT plan quality obtained using the DL approach is clinically acceptable. Moreover, the DL approach enables WBRT auto‐planning without the time‐consuming manual MLC shaping and target contouring.

## 1 Introduction

Metastatic brain cancer is the most common type of intracranial tumor.^1^ From common primary cancer sites such as lung, breast, and melanoma, brain metastasis occurs in 15% to 40% of the cancer patients.^2^, ^3^ The treatment of metastatic brain tumors depends on the number of metastatic tumors, extracranial tumor status, and performance status.^4^, ^5^, ^6^ Currently, whole‐brain radiation therapy (WBRT) is considered a well‐established treatment for patients with multiple brain metastases. For patients with resectable solitary brain metastases, surgical resection followed by adjuvant WBRT, which is a standard treatment with enhanced survival chances, demonstrated improved oncological outcomes.^4^, ^7^ Meanwhile, several prospective studies suggested stereotactic radiosurgery (SRS) as an effective treatment option for patients with three brain metastasis or less.^4^, ^8^, ^9^, ^10^ These studies, which were based on the fewer side effects and higher local control of SRS compared to WBRT, are inconclusive about the optimal treatment strategy for a small number of brain metastases, even though they have reported encouraging results. Additionally, compared to WBRT, the SRS approach has an added risk of intracranial progression outside the treatment field.^9^ However, a noninvasive treatment combining WBRT with SRS has demonstrated improved survival in cases of brain metastases.^11^, ^12^ Although several treatment approaches have been proposed, WBRT remains the cornerstone for the treatment of brain metastases.^6^, ^11^, ^12^

Because multiple brain metastases with or without peri‐tumoral edema can lead to various symptoms such as headache, nausea, vomiting, seizure, and visual field disorders,^6^, ^13^ we often encounter patients in urgent need of WBRT to alleviate the symptoms. However, radiation therapy requires multi‐step processes, including simulation computer tomography (CT) scan, normal organ contouring, gross tumor volume contouring, clinical target volume contouring, and radiation treatment planning (RTP). Therefore, depending on the complex workflow scheduling, radiation therapy can be delayed. It has recently been reported that deep‐learning techniques provide improved workflow for radiation therapy.^14^, ^15^, ^16^, ^17^ The outcomes of the contouring tasks performed by deep‐learning methods are improved consistency of contouring quality and reduced contouring time.^14^, ^15^, ^16^ Also, the automated RTP with deep learning provides shorter planning time with lower error rate.^17^

In this study, we investigate the feasibility of an automated RTP for WBRT, designed using deep‐learning techniques for predicting the multileaf collimator (MLC) shape bypassing the contouring processes. Further, this study compares the RTP results obtained by the deep‐learning approach with the results of the human‐driven RTP, which includes multiple steps of contouring and MLC shaping.

## 2 Materials and Methods

### 2.1 Datasets

Fifty‐five patients, treated with WBRT in our hospital, were chosen. They were treated with total doses of 30 Gy in 10–12 fractions. The treatment plans, which were designed in C‐Linac 2300 iX and VitalBeam (Varian Medical System, Palo, Alto, CA, USA) with a 120‐leaf MLC, had two lateral radiation static fields (gantry angles of 90° and 270°) with the treatment isocenter at the center between the eyes. The C‐spine region was excluded from the treatment plans to prepare a consistent dataset to be localized in the brain region as a target. In those 55 cases, 45 cases were assigned to the training and validation sets, while the remaining 10 cases were assigned to the test set to evaluate the plan quality for unknown cases.

**Fig. 1:**
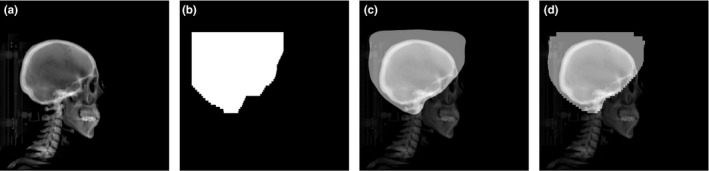
DRR images and MLC masks: (a) DRR‐reconstructed CT images, (b) mask image of manually optimized MLC field in white, (c) DRR image overlaid with MLC shape predicted by the deep learning, and (d) DRR image overlaid with a predicted MLC shape considering the discrete leaf size

The static MLC shapes, as reference were obtained from the manually optimized (MO) treatment plan files formatted in Digital Imaging and Communications in Medicine (DICOM). A dosimetrist optimized the MLC shape used in the treatment based on the CT image in the treatment planning system (TPS) of Eclipse (Eclipse 15.6, Varian Medical Systems, Palo Alto, CA, USA). The digitally reconstructed radiograph (DRR) image used in the deep learning (DL), shown in Fig. 1(a), was generated with Hounsfield unit range of (−100, 1000) from the CT image. The open‐field region collimated by MLC was converted into a white color mask image, as shown in Fig. 1(b). The MLC shape [in Fig. 1(c)] predicted by the DL process was finally converted to the real MLC shape [in Fig. 1(d)] with 120 leaf positions. The mask and DRR images were saved as portable network graphics (PNG) files with a single gray channel after pixel normalization in the range of 0–255 under the same scale for all cases. The pixel values of both DRR and MLC shape were normalized to one in the DL training procedure. A dataset that was 15 times larger than the reference was generated by image data augmentation via rotation, translation, shearing, and zooming with the nearest filling mode.

### 2.2 U‐net model and training

**Fig. 2:**
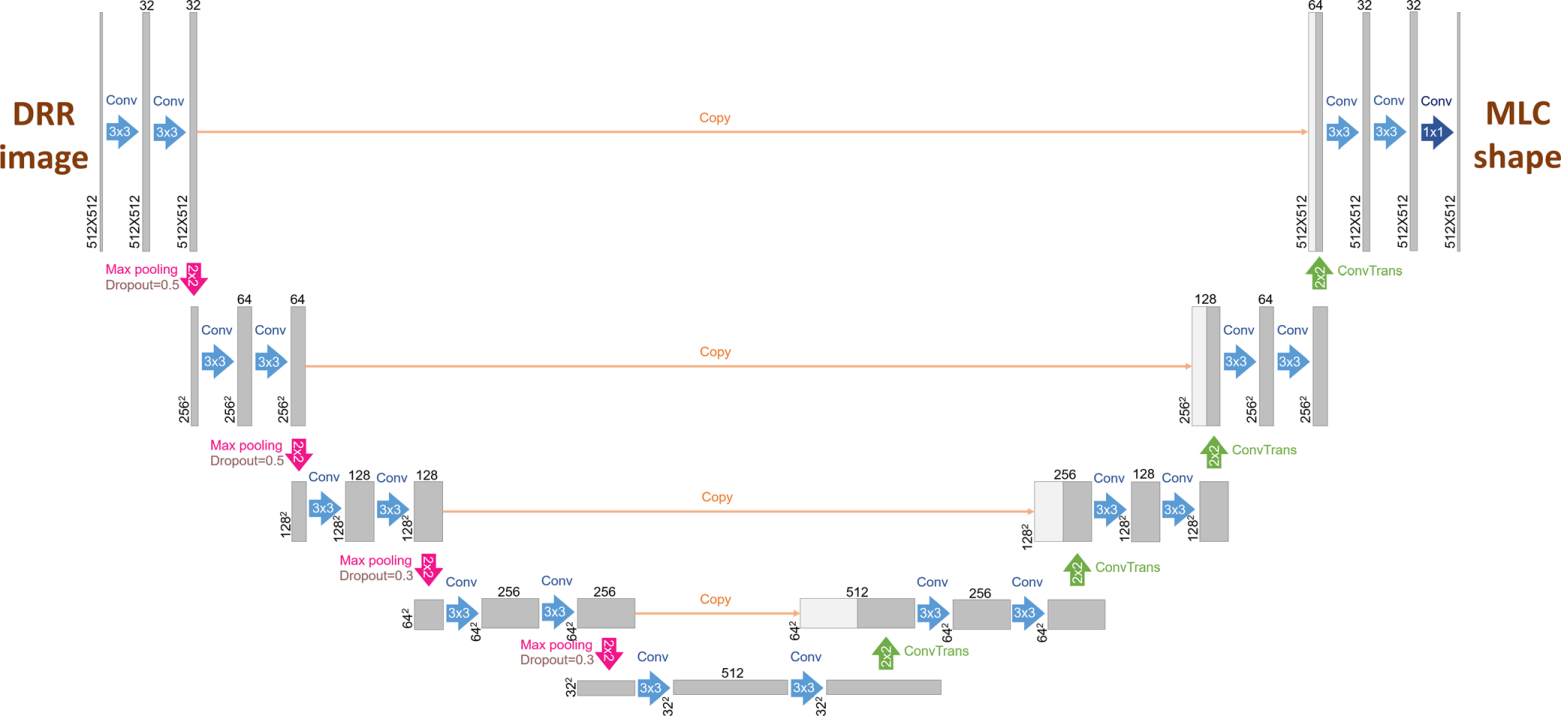
Architectures for the U‐net model using the input shape of DRR and the output shape of MLC. Each gray (light gray) box represents the (copied) multichannel feature map. The number of maps (map size) is denoted on the upper side (left‐side) of the box. The different operations are denoted as the arrows with different colors indicating different filter sizes

The DL framework, Tensorflow^18^ with Keras,^19^ was used for the automatic treatment planning of whole‐brain cases. This analysis used the convolution neural network (CNN) architecture called “U‐net,”,^20^ which is the convolutional encoder‐decoder network widely used to prevent resolution loss for the image segmentation. The shapes of the DRR in the input layer and MLC in the output layer were one‐to‐one connected via the U‐net modeling shown in Fig. 2. Both the input and output layers consisted of 512 × 512 voxels with a single channel. The network for the downsampling of feature maps contained two‐dimensional (2D) convolution (Conv2D) blocks and applied 3 × 3 filters with identical padding and rectified linear unit (ReLU) activation. Besides, max‐pooling (MaxPooling2D) to reduce the pixel size and random deactivation of the unit (dropout) were employed.^21^ For the first two convolution layer blocks, 50% dropout portions were achieved, and the dropout portion was 30% for the next two convolution layer blocks. The network for the upsampling of the feature map involved the inverse operation (Conv2DTranspose) of Conv2D and the concatenation process for better pixel localization regarding the input arrays. The output layer with the sigmoid activation was a 1 × 1 convolution with a single‐channel converted from a previous layer with 32 channels. The number of network parameters was 7.7 M. An adaptive moment estimation (Adam) optimizer with a learning rate of 5 × 10^‐5^ was applied. Dice similarity coefficient (DSC) as loss function was used for the similarity assessment between the input and predicted MLC shapes.^22^ The DSC is defined as −2|X_i_ ∩ X_p_+S| / (|X_i_|∪|X_p_|+S), where X_i_ (X_p_) is an input (predicted) tensor obtained from image pixel values, and S is the smoothness (in this study, S = 1). The training was performed up to 50 epochs until the DSC value was saturated in the learning curve with the validation samples. Further, DSC values were obtained in the ensemble learning for ninefold cross‐validation with samples of 40 training cases and five validation cases and were used for training reproducibility estimation. The DL training and test procedures were run under the following environment: Python 3.6.8, TensorFlow‐Gpu 1.14.0, Keras 2.2.4, and CUDA 10.1 with TITAN Xp 12 GB GPU (NVIDIA Corp., CA, USA) in the operating system Ubuntu 16.04.6 LTS.

### 2.3 MLC shape prediction performance

The automatic MLC shaping using the DL technique was evaluated. Dose‐volume‐histogram (DVH) curves constructed from the predicted MLC shape were compared with the MO results for dosimetric parameters of planning target volume (PTV) and organs‐at‐risk (OARs) such as lenses, eyes, and brainstem. The predicted MLC parameters on the 90° and 270° fields were imported to TPS. Dose calculations in given static MLC shaping were performed by the dose calculation algorithm of the analytical anisotropic algorithm (AAA) in Eclipse under the delivered monitor unit (MU) setting used in the previous treatment plan.^23^ For 10 test cases, excluded from the training and validation dataset, the consistency and deviations of DVH curves were used to assess the learning performance. Also, the relative dose covering of PTV (D_95%_, D_90%_, D_50%_, D_10%_, D_5%_, and D_max_) and the absolute maximum dose (D_max_) were calculated to estimate the target coverage and the OAR saving chances, respectively. Based on the statistical function library of SciPy v1.1.0 implemented on Python, the paired t‐test with a two‐tailed option was used to compare two data samples of MO and DL for DVH parameters. The significance level was set to *P* < 0.05 in this t‐test.

## 3 Results

**Fig. 3:**
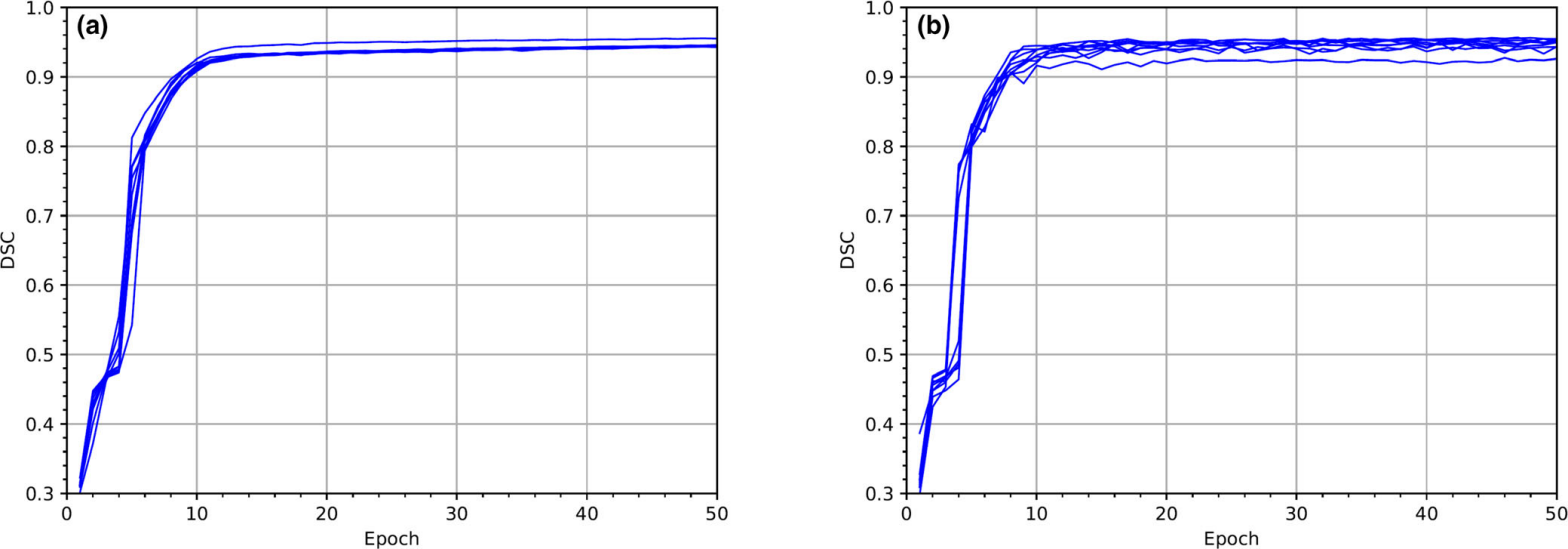
Learning curves for the ninefold cross‐validation with (a) training datasets and (b) validation datasets

The training and validation learning curves for ninefold cross‐validation are shown in Fig. 3. The training DSC curves saturate at 94.6 ± 0.4, where the errors were obtained from the standard deviation of the nine saturated DSC values while the validation DSC curves saturate at 94.7 ± 0.9. Above ten epoch, the validation DSC curves for different datasets show consistent and stable learning within the statistical variation in the saturation range.

**Fig. 4:**
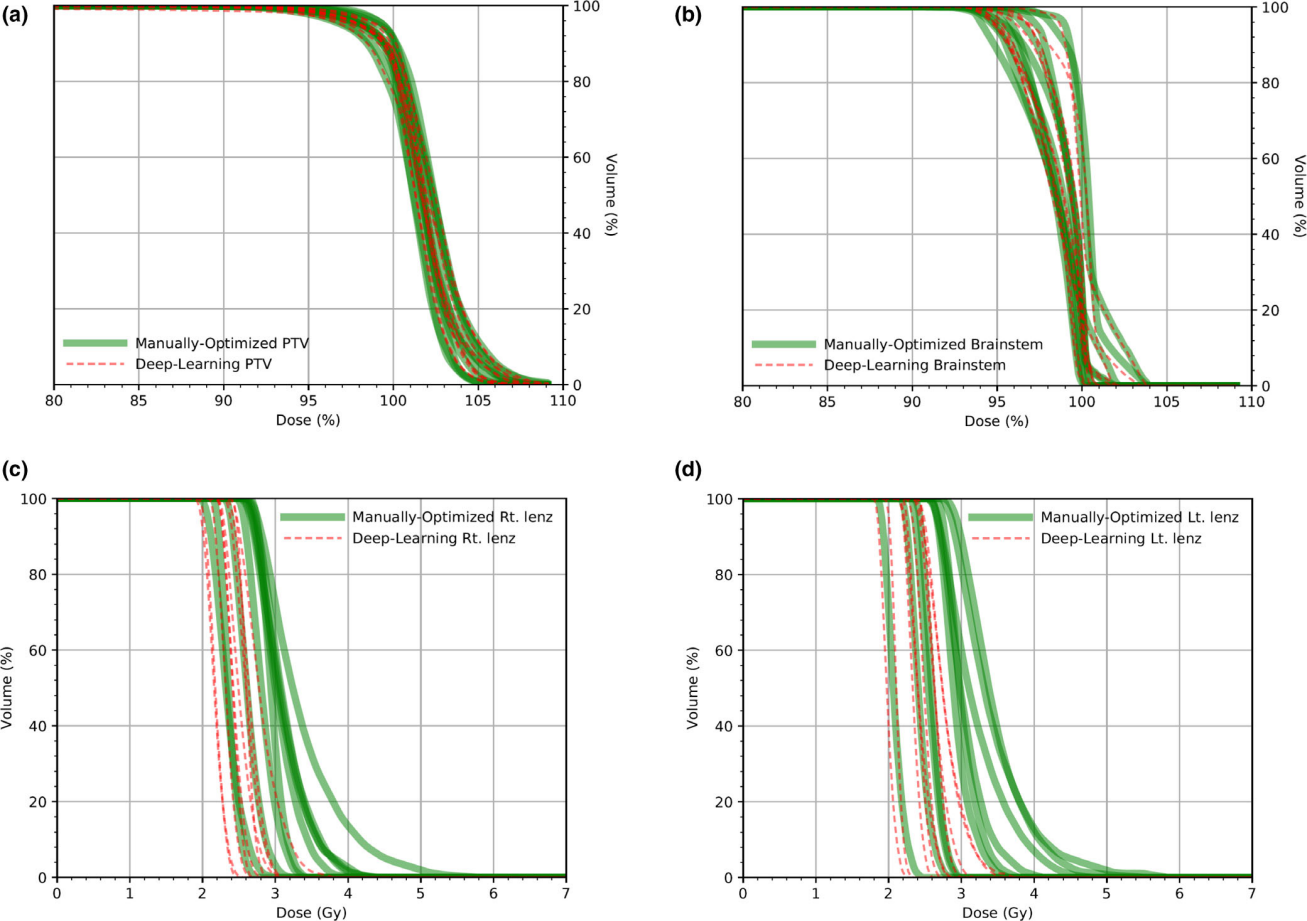
DVH comparisons evaluated with 10 test sample plans between the manually optimized plan (solid green line) and the deep‐learning plan (red dashed line) for (a) PTV, (b) Brainstem, (c) right lens, and (d) left lens

Ten DVH curves corresponding to 10 test samples obtained from the MO and the DL processes are depicted in Fig. 4 for (a) PTV, (b) brainstem, (c) right lens, and (d) left lens. The DVH curves of PTV and the brainstem for the DL approach are approximately identical with those for the MO approach. However, for the lens cases, DVH curves for DL shift toward the lower‐dose region compared to MO. The D_95%_ values of PTV and D_max_ values of OARs for these 10 cases are summarized in Table 1. The average D_95%_ value for PTV is (98.0 ± 0.7)% for DL and (98.3 ± 0.8)% for MO with uncertainties calculated from the standard deviation. DVH parameters of PTV except for D_max_ shows no significant difference, and D_max_ difference of PTV between MO and DL was 0.5% with *P* = 0.02. The OARs of the brainstem and eyes have approximately the same average D_max_ for both approaches except for the left eye case of patient 2 owing to anatomical difference. The average D_max_ values for both lenses as critical OARs are 2.9 Gy for DL and 3.9 Gy for MO. The lens D_max_ differences between MO and DL were approximately 1 Gy (*P* < 0.01).

**Table 1:** Clinical indices of PTV and OARs for ten whole‐brain patients in the manually optimized plan and the predicted plan by the deep‐learning technique. Averaged values with standard deviations and *P*‐values for 10 clinical indices were described

Structure	Planning method	Clinical indices	10 patients										Average ± SD	*P*‐value
			1	2	3	4	5	6	7	8	9	10		
PTV	MO	D_95%_ (%)	97.9	99.1	98.1	98.5	98.2	98.1	99.4	97.0	99.4	97.2	98.3 ± 0.8	0.135
	DL		98.0	97.6	98.0	98.5	98.0	98.0	99.1	96.9	98.9	97.4	98.0 ± 0.7	
	MO	D_90%_ (%)	99.4	100.1	99.7	99.5	99.5	99.4	100.2	98.5	100.2	98.8	99.5 ± 0.6	1.000
	DL		99.5	99.8	99.7	99.6	99.5	99.4	100.2	98.5	99.9	99.2	99.5 ± 0.5	
	MO	D_50%_ (%)	101.8	102.3	102.1	101.7	101.7	101.2	102.4	101.2	102.5	101.7	101.9 ± 0.5	1.000
	DL		101.9	102	102	101.8	101.7	101.4	102.5	101.4	102.2	101.7	101.9 ± 0.3	
	MO	D_10%_ (%)	104.5	105.3	104.2	103.8	104.3	103.1	105	103.0	104.9	103.4	104.2 ± 0.8	0.175
	DL		104.5	105.3	103.9	103.9	104.3	103.4	105.3	103.3	104.8	104.1	104.3 ± 0.7	
	MO	D_5%_ (%)	105.4	106.3	104.9	104.5	105.3	103.7	105.8	103.6	106.1	103.8	104.9 ± 1.0	0.209
	DL		105.4	106.3	104.5	104.7	105.4	104	106.1	103.9	105.9	104.6	105.1 ± 0.9	
	MO	D_max_ (%)	108.5	111	111.4	107.2	110.9	108.9	110.6	107.6	112.3	105.8	109.4 ± 2.1	0.017
	DL		109.1	111.5	111.8	107.6	111	109.1	110.8	108.2	112.1	107.4	109.9 ± 1.8	
Brainstem	MO	D_max_ (Gy)	32.2	30.1	35.6	26.3	31.9	30.3	31.1	30.4	32.8	32.0	31.3 ± 2.4	1.000
	DL		32.3	30.0	35.5	26.3	31.9	30.3	30.9	30.4	32.8	32.3	31.3 ± 2.4	
Rt. Lens	MO	D_max_ (Gy)	3.4	4.3	3.4	3.0	4.4	3.3	4.4	2.7	4.1	5.9	3.9 ± 0.9	0.004
	DL		2.7	2.5	2.9	2.5	3.8	2.8	3.1	2.7	3.1	3.1	2.9 ± 0.4	
Lt. Lens	MO	D_max_ (Gy)	4.2	3.7	3.8	2.5	4.9	3.0	3.0	2.9	5.9	5.5	3.9 ± 1.2	0.002
	DL		2.8	2.3	3.0	2.3	3.6	2.7	2.1	2.9	3.8	3.2	2.9 ± 0.6	
Rt. Eye	MO	D_max_ (Gy)	31.5	30.5	34.2	25.0	30.9	30.0	30.2	28.9	31.4	31.5	30.4 ± 2.4	0.030
	DL		29.9	25.7	33.6	24.5	30.7	29.9	29.7	27.1	30.9	30.7	29.3 ± 2.7	
Lt. Eye	MO	D_max_ (Gy)	31.7	30.1	34.0	25.0	31.2	29.9	29.9	29.4	31.6	31.6	30.4 ± 2.3	0.211
	DL		30.6	7.5	33.1	24.3	30.9	29.8	28.2	29.1	31.1	30.3	27.5 ± 7.4	

SD, standard deviations; PTV, planning target volume; MO, manually optimized; DL, deep‐learning.

**Fig. 5:**
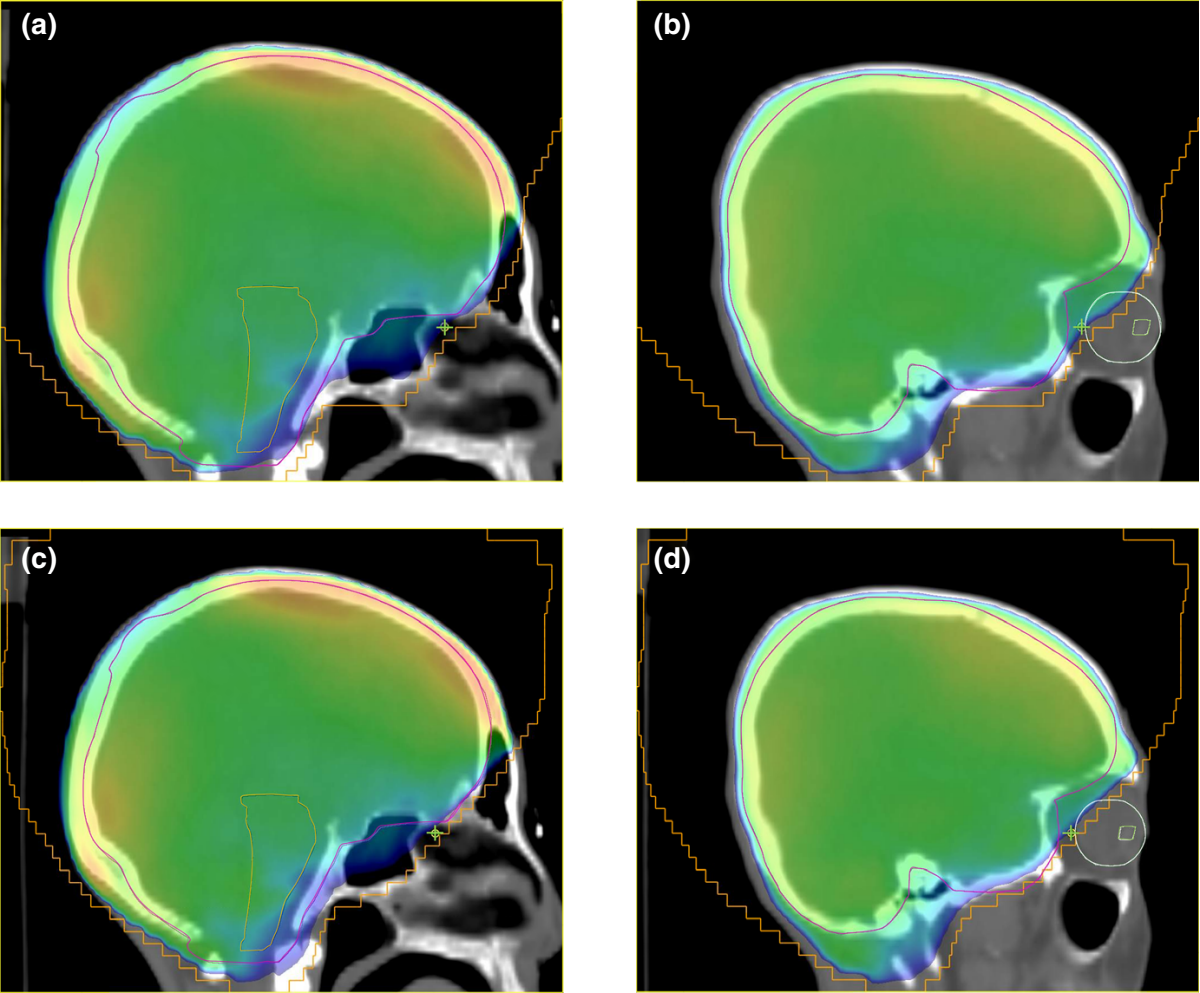
Example dose distributions of patient one on the planes with (a) isocenter for the manually optimized plan, (b) right lens for the manually optimized plan, (c) isocenter for the deep‐learning plan, and (d) right lens for the deep‐learning plan

An example case chosen from 10 test samples for the sagittal dose distributions is shown in Fig. 5. Upper two‐dose planes and lower two‐dose planes were obtained from the MO and the DL plans, respectively. Dose distributions in PTV and the brainstem, shown in Figs. 5(a) and 5(c), respectively, have no significant difference between both plans except for the dose in the eyeball regions shown in Fig. 5(b) and 5(d).

## 4 Discussion

The feasibility of the automatic MLC shape prediction planning using the DL technique was evaluated in this study with whole‐brain cases. To the best of our knowledge, this is the first study that predicts the MLC shape without additional contouring tasks using the U‐net DL model based on 2D DRR reconstructed from CT images.

For the usual whole‐brain planning, the shaping of MLC leaves does not follow the PTV shape, which means that the planning is designed to have sufficient open‐field space between the edge lines of the PTV and MLC fields in the superior brain region. Because the predicted MLC edge line is highly dependent on high contrast regions like bony structures in the DRR image, a question arises about the DL's decision of the borderline between the brain and other regions. Figures 5(c) and 5(d) show that some parts of the predicted MLC edge line are located more on the inner side than the lines shown in Figs. 5(a) and 5(b). This tendency of the field edge line may have originated from the DL property that assigns more weight to regions close to the bony structure. However, after dose calculations in given MLC shapes for the DL and MO, we observed, based on the two DVH curves in Fig. 4, that the DL treatment planning offered approximately similar plan quality to the reference MO plan. Moreover, Fig. 4 shows, and Table 1 confirms that the DL plan outperforms the MO plan in terms of lens dose reduction even though there is a small loss of approximately 0.3% in the dose coverage for PTV. Further, the distributions of the DL DVH curves verify that the DL method provides a consistent plan quality for different patient cases. The MO DVH curves are more spread than the DL DVH curves in general.

The training method using 2D DRR images directly predicts MLC leaf positions; thus, it enables quick planning without additional procedures such as target contouring and MLC shaping. Such a time‐saving planning procedure bypasses the time‐consuming routine jobs and results in the rapid treatment to alleviate patient pain. Also, the results of this study can be extended to the applications that use a 2D setup image (gantry angle of 90° or 270°) or a DRR image reconstructed from the cone‐beam CT scanning. If the MLC shape prediction based on patient setup images is available, the treatment procedure from the planning image acquisition to the radiation delivery to a patient will be significantly simplified. Also, implementations of other automatic techniques such as the plan import and the dose calculation are expected to accelerate the planning automation. This implies that the simulation for the treatment setup and the first treatment of a patient can be carried out simultaneously once the patient enters the treatment room. Because other studies^24^, ^25^ uses the fluence map estimation based on the treatment target to determine the aperture (or MLC) shape, those methods could obtain the accurate aperture shape. Our DL method is only available in cases with clear bone stures around targets such as whole brain without cervical spine involvement, although it has an advantange as being a more intuitive prediction for the MLC shape without fluence calcuations and target segmentations.

The limitations of this study leave considerable scope for future work in this field. A dose inhomogeneity with D_max_ = (109.8 ± 1.7)% up to 112.1% appeared in the outer region of the brain because only two static lateral fields were considered in this study. The automatic planning technique for dynamic MLC fields to mitigate hot dose regions would be a research target in future research. The inaccurate MLC shape prediction to small organs such as lenses should be also improved by the fine‐tuned DL modeling and the larger dataset. To achieve complete automated planning, MU estimation for a given prescribed dose is another exciting topic.

## 5 Conclusion

In this study, we evaluated training results using the DL technique with a dataset of 2D DRR images for whole‐brain cases. The predicted plan quality was clinically acceptable based on the DVH curves and was comparable with the result of the MO plan. This DL‐implemented planning without manual MLC shaping based on target contouring can help save time in the entire treatment process. It has the potential to improve the plan quality and enable rapid treatment in the whole‐brain radiation therapy.

## Conflicts of Interest

The authors have no relevant conflicts of interest to disclose.

## Author contribution

Jesang Yu and Youngmoon Goh wrote the manuscript together and conceived the research idea. Kye Jin Song prepared data files including the treatment plan and CT images, and performed calculations in the planning system. Youngmoon Goh developed the python codes for deep‐learning modeling and image preparations. Jungwon Kwak and Byungchul Cho verified the methods focused on the treatment planning. Jesang Yu and Su San Kim also clinically verified the plan results. Sang‐wook Lee and Eun Kyung Choi supervised the findings and provided the interpretations for this work. All authors discussed the results and commented on the manuscript.

## References

[1] Schouten LJ , Rutten J , Huveneers HA , Twijnstra A . Incidence of brain metastases in a cohort of patients with carcinoma of the breast, colon, kidney, and lung and melanoma. Cancer. 2002;94:2698–2705. 12173339 10.1002/cncr.10541

[2] Khuntia D , Brown P , Li J , Mehta MP . Whole‐brain radiotherapy in the management of brain metastasis. J Clin Oncol. 2006;24:1295–1304. 16525185 10.1200/JCO.2005.04.6185

[3] Tosoni A , Ermani M , Brandes AA . The pathogenesis and treatment of brain metastases: a comprehensive review. Crit Rev Oncol/Hematol. 2004;52:199–215. 10.1016/j.critrevonc.2004.08.006 15582786

[4] Brown PD , Jaeckle K , Ballman KV et al. Effect of radiosurgery alone vs radiosurgery with whole brain radiation therapy on cognitive function in patients with 1 to 3 brain metastases: a randomized clinical trial. JAMA. 2016;316:401–409. 27458945 10.1001/jama.2016.9839 PMC5313044

[5] Fritz C , Borsky K , Stark LS et al. Repeated courses of radiosurgery for new brain metastases to defer whole brain radiotherapy: feasibility and outcome with validation of the new prognostic metric brain metastasis velocity. Front Oncol. 2018;8:551. 30524969 10.3389/fonc.2018.00551 PMC6262082

[6] Franchino F , Rudà R , Soffietti R . Mechanisms and therapy for cancer metastasis to the brain. Front Oncol. 2018;8:161. 29881714 10.3389/fonc.2018.00161 PMC5976742

[7] Lamba N , Muskens IS , DiRisio AC et al. Stereotactic radiosurgery versus whole‐brain radiotherapy after intracranial metastasis resection: a systematic review and meta‐analysis. Radiat Oncol. 2017;12:106. 28646895 10.1186/s13014-017-0840-x PMC5483276

[8] Yamamoto M , Serizawa T , Shuto T et al. Stereotactic radiosurgery for patients with multiple brain metastases (JLGK0901): a multi‐institutional prospective observational study. Lancet Oncol. 2014;15:387–395. 24621620 10.1016/S1470-2045(14)70061-0

[9] Aoyama H , Shirato H , Tago M et al. Stereotactic radiosurgery plus whole‐brain radiation therapy vs stereotactic radiosurgery alone for treatment of brain metastases: a randomized controlled trial. JAMA. 2006;295:2483–2491. 16757720 10.1001/jama.295.21.2483

[10] Kocher M , Soffietti R , Abacioglu U et al. Adjuvant whole‐brain radiotherapy versus observation after radiosurgery or surgical resection of one to three cerebral metastases: results of the EORTC 22952–26001 study. J Clin Oncol. 2011;29:134–141. 21041710 10.1200/JCO.2010.30.1655 PMC3058272

[11] Sun H , Xu L , Wang Y et al. Additional radiation boost to whole brain radiation therapy may improve the survival of patients with brain metastases in small cell lung cancer. Radiat Oncol. 2018;13:250. 30563554 10.1186/s13014-018-1198-4 PMC6299519

[12] Andrews DW , Scott CB , Sperduto PW et al. Whole brain radiation therapy with or without stereotactic radiosurgery boost for patients with one to three brain metastases: phase III results of the RTOG 9508 randomised trial. Lancet. 2004;363:1665–1672. 15158627 10.1016/S0140-6736(04)16250-8

[13] Wong E , Rowbottom L , Tsao M et al. Correlating symptoms and their changes with survival in patients with brain metastases. Annals of Palliative Medicine. 2016;5:253–266. 27701879 10.21037/apm.2016.09.01

[14] Lustberg T , van Soest J , Gooding M et al. Clinical evaluation of atlas and deep learning based automatic contouring for lung cancer. Radiother Oncol. 2018;126:312–317. 29208513 10.1016/j.radonc.2017.11.012

[15] Havaei M , Davy A , Warde‐Farley D et al. Brain tumor segmentation with Deep Neural Networks. Med Image Anal. 2017;35:18–31. 27310171 10.1016/j.media.2016.05.004

[16] Song J , Samant R , Jay M et al. Whole brain radiotherapy improves survival outcomes in primary CNS lymphoma patients ineligible for systemic therapy. Support Care Cancer. 2020;28:5363–5369. 32140974 10.1007/s00520-020-05376-2

[17] Wang C , Zhu X , Hong JC , Zheng D . Artificial intelligence in radiotherapy treatment planning: present and future. Technol Cancer Res Treat. 2019;18:153303381987392 10.1177/1533033819873922 PMC6732844 31495281

[18] Keras deep learning library for python 2014. https://keras.io

[19] TensorFlow machine learning platform 2016. https://www.tensorflow.org

[20] Ronneberger O , Fischer P , U‐net BT . Convolutional networks for biomedical image segmentation. MICCIA. 2015;LNCS‐9351:234–241.

[21] Srivastava N , Hinton G , Krizhevsky A , Sutskever I , Salakhutdinov R . Dropout: a simple way to prevent neural networks from overfitting. J Mach Learn Res. 2014;15:1929–1958.

[22] Milletari F , Navab N , Ahmadi SA . V‐net: fully convolutional neural networks for volumetric medical image segmentation. IEEE. 2016 arXiv:1606.04797v1.

[23] Sievinen J , Ulmer W , Kaissl W . AAA photon dose calculation in Eclipse. Varian documentation RAD #7170B. 2005.

[24] Wang W , Sheng Y , Wang C et al. Fluence map prediction using deep learning models – direct plan generation for pancreas stereotactic body radiation therapy. Front Artif Intell. 2020;3:68. 10.3389/frai.2020.00068 PMC7861344 33733185

[25] Sheng Y , Li T , Yoo S et al. Automatic planning of whole breast radiation therapy using machine learning models. Front Oncol. 2019;9:750. 31440474 10.3389/fonc.2019.00750 PMC6693433

